# Long-Term Trends in Stroke Survivors Discharged to Care Homes: The South London Stroke Register

**DOI:** 10.1161/STROKEAHA.119.026618

**Published:** 2019-11-06

**Authors:** Amanda Clery, Ajay Bhalla, Alessandra Bisquera, Lesli E. Skolarus, Iain Marshall, Christopher McKevitt, Anthony Rudd, Catherine Sackley, Finbarr C. Martin, Jill Manthorpe, Charles Wolfe, Yanzhong Wang

**Affiliations:** 1 From the Department of Population Health Sciences, School of Population Health and Environmental Sciences (A.C., A. Bhalla, A.B., L.E.S., I.M., C.M., A.R., C.S., F.C.M., C.W., Y.W.), King’s College London, United Kingdom; 2 Department of Ageing and Health, Guy’s and St Thomas’ NHS Foundation Trust, London, United Kingdom (A. Bhalla, A.R.); 3 Department of Neurology, University of Michigan, Ann Arbor (L.E.S.); 4 National Institute for Health Research Policy Research Unit in Health and Social Care Workforce, King’s College London, London, United Kingdom (J.M.); 5 National Institute for Health Research Biomedical Research Centre, Guy’s and St Thomas’ NHS Foundation Trust, London, United Kingdom (C.W., Y.W.); 6 National Institute for Health Research Collaboration for Leadership in Applied Health Research and Care South London, United Kingdom (C.W., Y.W.).

**Keywords:** epidemiology, health services, London, nursing homes, risk factors, stroke

## Abstract

Care homes provide care to many stroke survivors, yet little is known about changes in care home use over time. We aim to determine trends in discharge to care homes, to explore the characteristics of stroke survivors over time (1995–2018), and to identify the associations between these characteristics and discharge to care homes poststroke.

**Methods—:**

Using data from the South London Stroke Register between 1995 and 2018, we estimated the proportions discharged to care homes and their characteristics over time, assessed by tests for trends. Multivariable logistic regression models were built to assess the associations between their characteristics and discharge destination.

**Results—:**

Of 4172 stroke survivors, 484 (12%) were discharged to care homes. This proportion has decreased from 24% in 1995 to 2000 to 5% in 2013 to 2018. The mean age of those discharged to care homes has increased over time, from 73 to 75 (*P*<0.001). Among stroke survivors discharged to a care home, the proportion with a prestroke Barthel Index <15 has also increased over time from 7% to 21% (*P*=0.027), while the proportion with a 7-day poststroke Barthel Index <15 remains largely unchanged over time (93% in 1995–2000, 90% in 2013–2018). The characteristics most strongly associated with discharge to care homes were (odds ratio [95% CI]) age (1.05 [1.04–1.07] per year), stroke subtype (hemorrhagic; 0.64 [0.43–0.95]), stroke severity (Glasgow Coma Scale score, <13; 1.67 [1.19–2.35]), failed swallow test at admission (1.65 [1.20–2.25]), 7-day poststroke Barthel Index <15 (3.58 [2.20–6.03]), and a longer hospital stay (1.02 [1.02–1.03] per day).

**Conclusions—:**

Over >20 years, there has been an 80% reduction in the proportion of stroke survivors discharged to care homes, influenced by changes in the demographics, disability, and stroke care in the underlying stroke population. In those moving to care homes, the level of poststroke disability remains high, requiring continued attention and investment.

## Abstract 2

Supplemental Digital Content is available in the text.

The health of care home residents throughout high-income countries is poor with many experiencing high levels of dependency, cognitive impairment, incontinence, and long-term health conditions.^1,2^ Residents who have had a stroke share these characteristics and have some of the highest levels of need.^3,4^

Estimates from the United States and United Kingdom suggest that 15% to 18% of care home residents >65 years of age have had a stroke.^5,6^ The UK Sentinel Stroke National Audit Programme reported that in 2017 to 2018, 10.9% of stroke patients nationally were discharged to a care home directly following the episode of care prompted by their stroke.^7^

Where to discharge a patient after stroke is a difficult decision to make for patients, their family, and health and care professionals alike.^8^ Returning home after their stroke is an important outcome for patients, and this is supported, for example, by increasing emphasis on rehabilitation in the community.^9^ The Stroke Action Plan for Europe 2018 to 2030 set targets for increased early supported discharge and improved community-based care.^9^ While reintegrating stroke survivors into the community is a priority, discharge to a care home may be the best option for those with complex health needs or limited support.^10^ This option is still a commonly taken one, the costs in the United Kingdom either being met by local government, the National Health Service, or paid for by individuals themselves.^11^

As populations age globally, the burden of stroke continues to increase.^12^ Increasing age is one of the strongest predictors for discharge to care homes.^13^ The aims of this study are to explore how the characteristics of stroke survivors discharged to care homes have changed over time (1995–2018) in a multiethnic, inner-city population, in the context of changing stroke care both in hospitals and the community and to identify the associations between these characteristics and discharge to care homes poststroke.

## Methods

The raw data for this study contain both personably identifiable and confidential clinical data. The participants of the study did not consent to sharing the information publicly, and our ethical approvals require strict information governance procedures. Requests for data access for academic use should be made to the South London Stroke Register (SLSR) team, where data will be made available subject to academic review and acceptance of a data-sharing agreement.

### Study Population

The SLSR is an ongoing, prospective, population-based register that has recorded all first-ever stroke patients since January 1, 1995, in a defined geographic area within Lambeth and Southwark, inner-city South London, United Kingdom. At the 2011 UK Census, the source population was 357 308, with 56% white, 25% black, and 18% other ethnic groups.^14^ Stroke is defined according to the World Health Organization definition.^15^ Cases are ascertained from hospital records, general practitioners, community healthcare professionals, and death certificates. Follow-up data are collected for patients at 3 months and annually poststroke. Details of data collection are described elsewhere.^16^

### Study Inclusion and Exclusion Criteria

Patients registered in the SLSR who had their first-ever stroke between January 1, 1995, and December 31, 2018, were included in this study. Those who were not admitted to hospital as a result of their stroke, who were already living in a care home at the time of stroke, or who died before discharge were excluded.

The definition of care home for this study includes both residential and nursing homes. Legally, these are both care homes in the English context, and many care homes are jointly registered, offering residential and nursing care together. Both provide accommodation and long-term care to residents, but nursing homes additionally provide 24-hour nursing cover from a registered nurse.^17^ Most care home residents live there permanently, although a small proportion are short-term residents intending to return home. Anyone who was discharged from the hospital to a care home or living at a care home by their 3-month follow-up was defined as having been discharged to a care home.

### Characteristics

Data on the following characteristics were evaluated, detailed elsewhere,^18^ including the following:

Sociodemographic characteristics: year of stroke, age at time of stroke, sex, ethnicity (white, black, and other), and prestroke living arrangements (alone or with other people).

Acute-phase clinical characteristics: stroke subtype (ischemic or hemorrhagic), Glasgow Coma Scale score at the time of maximum impairment (3, coma; 15, fully awake),^19^ swallow test result on admission to hospital (pass or fail), incontinence in hospital poststroke, Barthel activities of daily living index (Barthel Index [BI]) prestroke, BI 5 to 10 days poststroke (0, severe disability; 20, independent),^20^ and National Institutes of Health Stroke Scale at the time of maximum impairment (0, no stroke symptoms; 42, severe stroke; note: this variable was only collected from 2004 onward).

Acute-phase process of care: admission to a stroke unit, brain imaging (computed tomography or magnetic resonance imaging scans), length of stay in hospital (on any ward type, in days), and thrombolysis in those with an ischemic stroke (note: this variable was only collected from 2004 onward).

Prestroke risk factors: hypertension, transient ischemic attack, atrial fibrillation, myocardial infarction, diabetes mellitus, smoking status (never, ex-smoker, or current smoker), and alcohol consumption (defined as drinking >1 unit per week).

### Statistical Methods

Stroke survivors were grouped into 4 cohorts based on their year of stroke: 1995 to 2000, 2001 to 2006, 2007 to 2012, and 2013 to 2018. For each cohort, the proportion discharged to care homes was calculated, with a 95% CI. A test for trend was conducted to compare the proportions over the 4 cohorts.

For each cohort, characteristics were summarized using counts and percentages for categorical variables and means and SDs for continuous variables. Nonnormal continuous variables were summarized using medians and interquartile ranges. Changes over time (across the 4 cohorts) in the proportions with these characteristics were assessed using χ^2^ or Fisher exact tests where sample size was small, for categorical variables. Continuous variables were assessed using ANOVA for normal variables or Kruskal-Wallis tests for nonnormal. Missing data in the outcome and in all variables were also assessed (Tables I and II in the online-only Data Supplement). These trends were also tested in the whole population of stroke survivors in the SLSR (Table III in the online-only Data Supplement).

All characteristics were also assessed for associations with discharge to care homes (care home versus own home). This binary outcome was used in univariable logistic regression analyses to summarize the crude associations between each variable and discharge destination with odds ratios and 95% CIs. Likelihood ratio tests were conducted between each regression model and the null model to obtain *P*. Associations were assessed in a multivariable model adjusted for all other variables in the model. For variables with ≥3% missing data, missing values were treated as a separate category. These models were also built in the 4 separate cohorts as a subgroup analysis (Tables IV through VII in the online-only Data Supplement).

All analyses were conducted in the statistical software R, version 3.5.1, or later.

### Ethics

All patients or relatives gave written informed consent to participate in the study. Ethical approval for the study was obtained from the ethics committees of King’s College Hospital, Queen’s Square, Westminster Hospital, and Guy’s and St Thomas’ NHS Foundation Trust (No. EC01 020).

## Results

**Figure 1.**
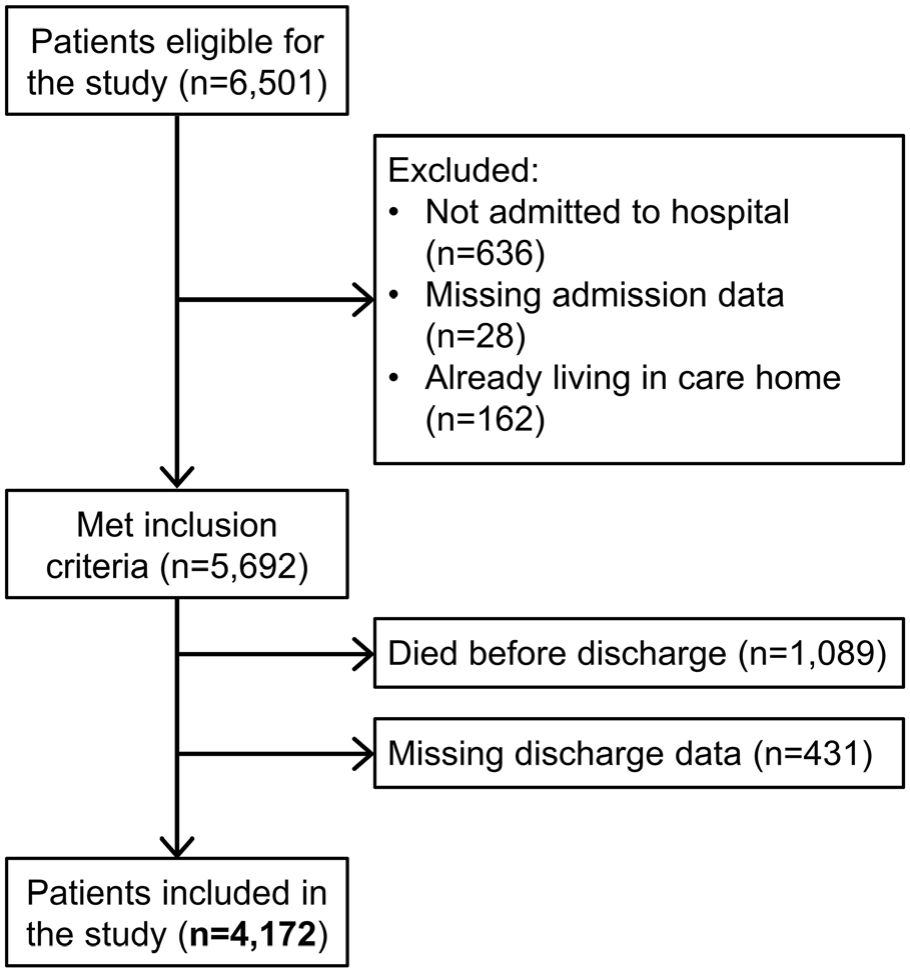
Flowchart of study participants.

Six thousand five hundred one patients in the SLSR had a first-ever stroke between January 1, 1995 and December 31, 2018. After applying exclusion criteria, 4172 stroke survivors with known discharge status were identified (Figure 1). Of these, 484 (12%) were discharged to care homes, 3441 (83%) to their own homes, and 247 (6%) to other destinations (including community hospitals and sheltered housing/rental housing with telecare alarm services and other modifications).

**Figure 2.**
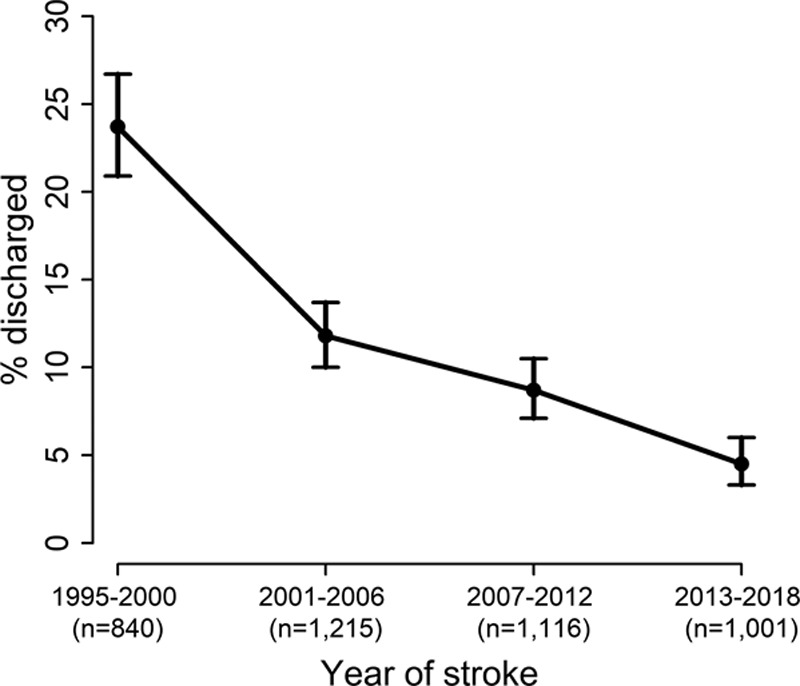
Proportion of each cohort discharged to care homes.

Figure 2 shows that there is a decreasing trend in the proportion of stroke survivors discharged to care homes over time (*P*<0.001). In 1995 to 2000, 24% (95% CI, 21%–27%) of stroke survivors were discharged to care homes, compared with 12% (10%–14%) in 2001 to 2006, 9% (7%–11%) in 2007 to 2012, and only 5% (3%–6%) in 2013 to 2018, summarized in Table 1. The proportion of those who died before hospital discharge has also decreased over time, from 30% in 1995 to 2000 to 14% in 2013 to 2018. Consequently, the proportion of survivors discharged to their own home has increased from 72% to 92% over a >20-year time span.

**Table 1.**
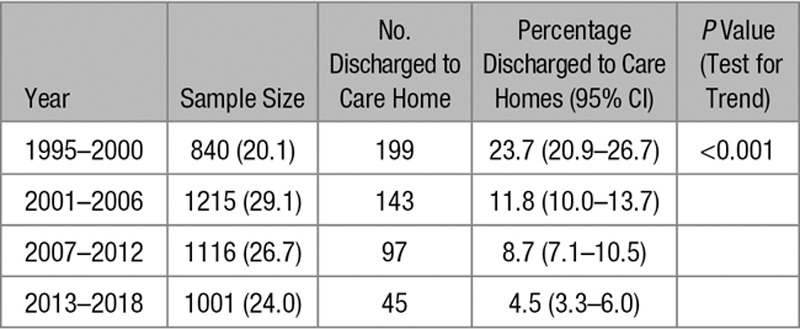
The Proportion of Each Cohort Discharged to Care Homes

### Trends in Characteristics of Stroke Survivors Discharged to Care Homes Over Time

Table 2 shows the characteristics of the 484 stroke survivors discharged to care homes, according to the year of stroke. Mean age at the time of stroke has increased over time from 73 to 80 years (*P*<0.001) in the 2007 to 2012 cohort, although has decreased to 75 in more recent years. While age of stroke survivors discharged to care homes has increased over time, there has been no change in the age of stroke survivors in the whole population (Table III in the online-only Data Supplement). Trends in the proportions of sex, ethnicity, and prestroke living arrangements of stroke survivors discharged to care homes remain unchanged over time. Comparatively, in the whole population, there has been a decline in the proportion of white patients and an increase in black and other ethnic groups, as well as a decline in the proportion who lived alone prestroke.

**Table 2.**
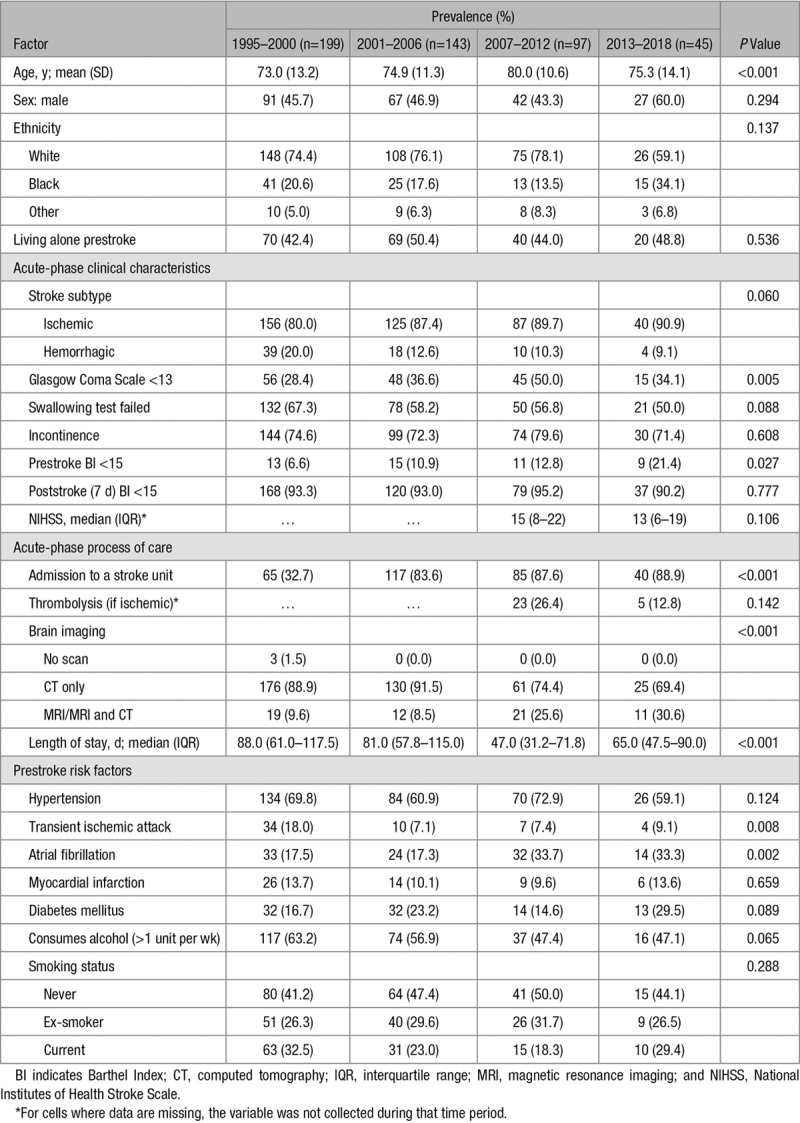
The Characteristics of Stroke Survivors Discharged to Care Homes, Stratified by Year of Stroke

There has been a decrease in the proportion of hemorrhagic strokes from 1995 to 2018 in both those discharged to care homes and the whole population (from 20% to 9%, *P*=0.060 and from 20% to 17%, *P*<0.001, respectively). Despite this, there was a substantial increase in the proportion of stroke survivors discharged to care homes with a more severe stroke (Glasgow Coma Scale score, <13; from 28% to 50% between 1995 and 2012 but decreased to 34% in 2013–2018; *P*=0.005). Similarly, there was also a slight increase in severe stroke in the whole population (from 15% to 16% between 1995 and 2018; *P*=0.037; Table III in the online-only Data Supplement). A slight decrease in the National Institutes of Health Stroke Scale was also observed in the most recent cohort, although not significant (from 15 to 13; *P*=0.1) as compared with a similar decrease in the whole population of stroke survivors (from 5 to 4; *P*<0.001).

We found no change in the proportion discharged to care homes over time who were disabled (BI<15) 7 days poststroke (from 93% to 90%; *P*=0.777). Comparatively, we found a decrease in the proportion of the whole population with poststroke disability over time (from 64% to 44%; *P*<0.001).

Access to in-hospital stroke care for those discharged to care homes has improved over time. More patients were admitted to stroke units (from 33% to 89%; *P*<0.001), more underwent a magnetic resonance imaging scan compared with a computed tomography alone (from 10% to 31%; *P*<0.001), and length of stay in hospital decreased over time (median, from 88 to 65 days; *P*<0.001). Transient ischemic attack (from 18% to 9%; *P*=0.008) as a prestroke risk factor has decreased over time, whereas recorded atrial fibrillation has almost doubled in 20 years (from 18% to 33%; *P*=0.002). These results are comparable to the trends seen in the whole population.

### Associations With Discharge to Care Homes

Comparing those who were discharged to their own home and to care homes, we identified several factors associated with discharge to care homes. Because of missing data in some variables, the final multivariable model analyzed 3341 patients, 391 (12%) of whom were discharged to care homes.

Those characteristics associated with discharge to care homes include (odds ratio [95% CI]) increasing age (1.05 [1.04–1.07] per year increase; *P*<0.001), male sex (0.72 [0.53–0.97] for women compared with men; *P*=0.029), stroke subtype (0.64 [0.43–0.95] for hemorrhagic compared with ischemic stroke; *P*=0.031), Glasgow Coma Scale score <13 (1.67 [1.19–2.35]; *P*=0.003), failed swallow test (1.65 [1.20–2.25]; *P*=0.002), incontinence (1.91 [1.38–2.65]; *P*<0.001), 7-day poststroke BI <15 (3.58 [2.20–6.03]; *P*<0.001), and increasing length of stay in hospital (1.02 [1.02–1.03] per day; *P*<0.001), summarized in Table 3. The subgroup analyses showed largely similar results for each of the 4 cohorts compared with the overall cohort (Tables IV through VII in the online-only Data Supplement). In each cohort separately, age and length of hospital stay were most strongly associated with discharge to care homes. However, as time progressed, we show that stroke severity (Glasgow Coma Scale score) and poststroke disability (BI) became more important in predicting discharge destination than the earliest cohort (1995–2000) except for the most recent cohort (2013–2018) where the number of patients discharged to care homes (n=39) is likely to be too small to provide sufficient power for analysis.

**Table 3.**
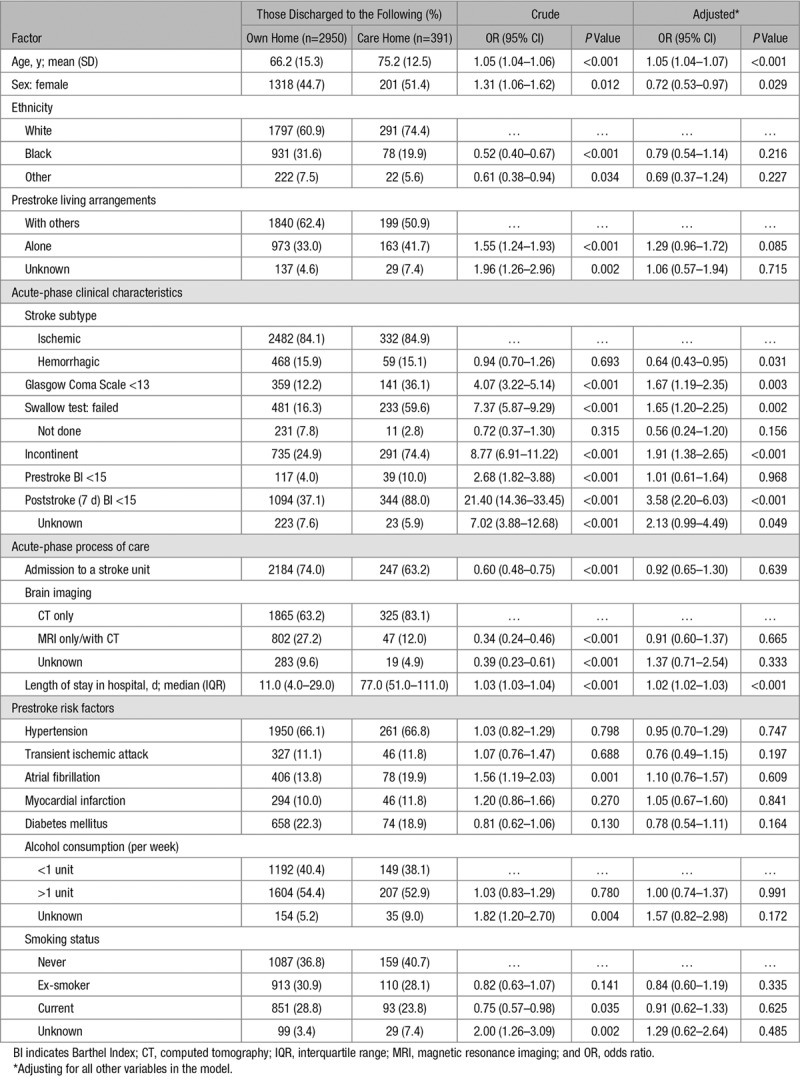
The Associations Between Demographic and Stroke Characteristics and Discharge Destination Poststroke

## Discussion

Over the course of 24 years from 1995 to 2018, there was an 80% reduction in the proportion of stroke survivors discharged to care homes. This decline in stroke survivors moving to care homes might be explained by changes in demographics and stroke severity in the whole population of stroke survivors, changes to acute stroke care, as well as poststroke care.

In the whole population of stroke survivors in the study area, we have shown a decline over time in the proportion with poststroke disability—one of the factors most strongly associated with discharge to care homes. Therefore, this decline in poststroke disability over time may play a part in the steep decline in those discharged to care homes over time.

In addition, we found a decline in the proportion of stroke survivors living alone prestroke in the whole population. Our study shows evidence that those living alone prestroke have higher odds of discharge to care homes compared with those who live with others prestroke, in agreement with previous research.^21–23^ This change in living arrangement in the study population in combination with its association with discharge destination may also explain the decline seen in care home discharge.

Other explanations include the changes in stroke care. Over the course of the study period, the study area has undergone major changes in stroke care. We have shown evidence that admissions to stroke units have improved substantially over time. More recently, thrombolysis and thrombectomy have also been introduced into hospital care in the study area. It is possible that improvements in quality and access to care has led to better rates of recovery and, therefore, fewer people being discharged to care homes.

After discharge from the hospital, the options for stroke care have also changed, which may account for some of the decline in discharges to care homes. There has been an increased emphasis on early supported discharge in the study area, which has been shown to reduce discharge to care homes for a select group of patients, but this is unlikely to be appropriate for those with the most severe strokes and disability.^24^ This increased availability of community support would enable those who previously might have moved to care homes to now return to their own home.

Beyond this study, the supply of care home places in the United Kingdom has fallen by around 20% from over 550 000 beds in 1995 to 450 000 in 2017.^11^ There is also evidence for a shift from the provision of high-cost nursing homes to lower cost residential homes.^11^ These reductions in the funding and availability of care homes may be an important factor leading to fewer numbers being discharged to care homes poststroke.

As the model of stroke care changes over time, it is important to understand the needs of those accessing the different forms of care. While there has been a decline in discharge to care homes, those who are moving to care homes continue to have the highest levels of disability. Around 75% of stroke survivors discharged to care homes are incontinent and 60% have swallowing difficulties at the time of stroke. Ninety percent are severely disabled at 7 days poststroke. These proportions have remained consistently high over time. The magnitude of care needs is notable; those with a poststroke BI <15 have 3.5× the odds of discharge to a care home compared with those with greater independence. Our results highlight the complex, time-intensive care needs of stroke survivors discharged to care homes. Determining the optimal way to care for this vulnerable population is important.

### Limitations

Of our stroke survivors, 9.4% had missing discharge destination. We identified some differences in those with missing and nonmissing discharge data (Table I in the online-only Data Supplement), which suggested that those with missing discharge destination have poorer functional outcomes. These characteristics are more similar to those discharged to care homes; therefore, it is possible that this was the destination for some patients with missing discharge information. Consequently, we may be undercounting the number of patients discharged to care homes in the study, especially in the earlier years between 1995 and 2000.

Because of high levels of missing data in variables such as cognitive impairment and dementia, we were unable to assess these in this study, although these are likely to be important contributors to the decision to discharge to care homes.

Finally, because of sample size restrictions, this study combines nursing and residential care. In England, these 2 forms of care are often also provided in the same establishment. Those with nursing care are likely to have poorer health than those in residential homes; so including all homes may have diluted some of the effects of nursing homes.

Lastly, our data on poststroke disability were measured at 5 to 10 days poststroke. Between this time point and discharge, a patient’s disability score may have changed, therefore, influencing the associations identified in this study. However, the 5- to 10-day BI is likely to be a reasonable indicator for a patient’s disability at discharge.

### Implications

This study uses the SLSR, which is unique for providing long-term trends in stroke patients. We have shown a decline in the proportion of stroke survivors discharged to care homes over more than a 20-year period. We have provided evidence for changes in demographics, poststroke disability, and acute stroke care that may help explain this, although factors beyond the scope of this study including community care at home and supply of care home beds in the study area are likely to also play a role in the decline in discharge to care homes seen. Under the Care Act 2014, English local government is responsible for shaping the care market, and our data may be helpful to their planning. We have highlighted that those moving to care homes continue to have the highest levels of need, which requires continued investment in equipment and staff to promote the quality of life and well-being of residents with stroke-related and other needs. Finally, as more stroke survivors are being discharged to their own homes, community investment in resources such as home care, carer support, and general accessibility could also be considered.

## Acknowledgments

We would like to express our thanks to all the participants of the South London Stroke Register and their families. We also thank the fieldworkers who have collected the data since 1995. Conceptualization was done by Drs Wolfe and Wang, methodology by A. Clery and Dr Wang, statistical analysis by A. Clery, supervision by Dr Wang, initial draft of paper by A. Clery, revision and review of paper by all, and funding by Dr Wolfe, Dr Sackley, J. Manthorpe, Dr McKevitt, A. Rudd, Dr Marshall, Dr Wang, and Dr Martin.

## Sources of Funding

This study was funded/supported by a Dunhill Medical Trust grant (R537/0217), the National Institute for Health Research (NIHR) Biomedical Research Centre at Guy’s and St Thomas’ NHS Foundation Trust and King’s College London, NIHR Collaboration for Leadership in Applied Health Research and Care South London, and the NIHR Policy Research Unit in Health and Social Care Workforce.

## Disclosures

None.

## Supplementary Material
