# Supplementary material for: Long-Term Trends in Stroke Survivors Discharged to Care Homes: The South London Stroke Register
Source: Stroke. 2019 Nov 6;51(1):179–85. doi: 10.1161/STROKEAHA.119.026618 (PMC6924949; doi:10.1161/STROKEAHA.119.026618)
Supplement: Supplementary file 1 [file str-51-179-s001.pdf]

SUPPLEMENTAL MATERIAL

Long-term trends in stroke survivors discharged to care homes: The South London Stroke Register

Amanda Clery, MSc[1], Ajay Bhalla, MD[1,2], Alessandra Bisquera, MSc[1], Lesli E. Skolarus, MD[1,3], Iain Marshall, PhD[1], Christopher McKevitt, PhD[1], Anthony Rudd, FRCP (Lond) [1,2], Catherine Sackley, PhD[1], Finbarr C. Martin, MD[1], Jill Manthorpe, MA[4], Charles Wolfe, MD[1,5,6], Yanzhong Wang, PhD[1,5,6]

1 School of Population Health and Environmental Sciences, King's College London, London, United Kingdom **2** Guy’s and St Thomas’ NHS Foundation Trust, London, United Kingdom **3** Department of Neurology, University of Michigan, Ann Arbor, USA **4** National Institute for Health Research (NIHR) Policy Research Unit in Health and Social Care Workforce, King’s College London, London, United Kingdom **5** NIHR Biomedical Research Centre, Guy's and St Thomas' NHS Foundation Trust and King's College London, London, United Kingdom, **6** NIHR Collaboration for Leadership in Applied Health Research and Care (CLAHRC) South London, London, United Kingdom

Supplemental Methods

Missing data (Tables I and II)

Missing data in the outcome (discharge destination) were summarised by categorising the outcome into missing or non-missing. The characteristics of stroke survivors were then summarised, stratified by this variable (Table I). Categorical variables were summarised using counts and percentages and chi-squared tests. Continuous variables were summarised using means, standard deviations and t-tests or medians, interquartile ranges and Mann-Whitney U-tests tests if non-normal.

The proportion of each variable that was missing was then also summarized, stratified by discharge destination (Table II). Similar statistical methods were used, as described above, to summarise the data.

Trends in the characteristics of the whole population of stroke survivors (Table III)

The statistical methods used here are similar to those used to assess the trends in stroke survivors discharged to care homes in the main results. Chi-squared tests for trend were used to summarise the trend over time in categorical variables and ANOVA for continuous variables (or Kruskal-Wallis if non-normal).

The associations between characteristics and discharge destination post-stroke, analysed separately for the 4 cohorts: 1995-2000, 2001-2006, 2007-2012, 2013-2018 (Tables IV-VII)

The data were divided into the 4 cohorts. In each cohort, missing data in all the variables were re-assessed. Any variables with >3% missing data had an additional missing data category added for analysis. Sample size was restricted for a complete-case analysis. Multivariable logistic regression models were built for all the variables, adjusting for year of stroke, age, sex, ethnicity, pre-stroke living arrangement, type of stroke, stroke severity (GCS), swallow test results, incontinence, pre-stroke BI, post-stroke (7d) BI, admission to a stroke unit, brain imaging, and length of stay in hospital. Results were summarised with odds ratios and 95% confidence intervals. Wald tests were used to obtain p-values for the associations.

Supplemental Results

Table I. The characteristics of stroke survivors, stratified by missingness in outcome data

Factor	Missing (N=431)	Non-missing (N=4,172)	P-value
Year of stroke			<0.001
1995-2000	297 (68.9)	840 (20.1)	
2001-2006	75 (17.4)	1215 (29.1)	
2007-2012	41 (9.5)	1116 (26.7)	
2013-2018	18 (4.2)	1001 (24.0)	
Age (mean (SD))	69.7 (15.5)	67.6 (15.3)	0.005
Sex: Male	216 (50.1)	2255 (54.1)	0.131
Ethnicity			<0.001
White	318 (76.1)	2582 (62.6)	
Black	82 (19.6)	1246 (30.2)	
Other	18 (4.3)	294 (7.1)	
Living alone pre-stroke	61 (36.7)	1404 (36.0)	0.919
**Acute phase clinical characteristics**			
Stroke subtype: Haemorrhagic	111 (26.6)	656 (15.8)	<0.001
Glasgow coma scale <13	128 (32.9)	632 (15.6)	<0.001
Swallowing test failed	159 (47.6)	913 (24.0)	<0.001
Incontinence	177 (50.9)	1291 (31.9)	<0.001
Pre-stroke BI<15	22 (5.7)	200 (4.9)	0.596
Post-stroke (7d) BI<15	115 (60.5)	1805 (48.2)	0.001
NIHSS (median (IQR))	6 (4-15)	5 (3-9)	0.055
**Acute phase process of care**			
Admission to a stroke unit	130 (32.7)	2991 (71.8)	<0.001
Thrombolysis (if ischaemic)	12 (25.5)	303 (13.3)	0.027
Brain imaging			<0.001
No scan	30 (7.9)	19 (0.5)	
CT only	321 (84.0)	2695 (71.4)	
MRI only/with CT	31 (8.1)	1060 (28.1)	
Length of stay, days (median (IQR))	16 (6-38)	14 (5-43)	0.803
**Pre-stroke risk factors**			
Hypertension	178 (54.3)	2718 (66.3)	<0.001
Transient ischemic attack	28 (8.2)	457 (11.2)	0.109
Atrial fibrillation	63 (18.4)	627 (15.4)	0.164
Myocardial infarction	30 (8.7)	416 (10.2)	0.422
Diabetes	66 (19.1)	913 (22.3)	0.188
Consumes alcohol (>1 unit per week)	177 (55.7)	2198 (56.8)	0.735
Smoking status			<0.001
Never	141 (37.0)	1543 (38.8)	
Ex-smoker	64 (16.8)	1244 (31.3)	
Current	176 (46.2)	1192 (30.0)	

Missingness in the outcome, discharge destination, is 9.4%. We constructed a binary variable to indicate whether the outcome was missing (431 patients) or non-missing (4,172). Table I shows the differences in characteristics between these two groups. Compared to those with non-missing outcome, those with missing data were more likely to: be from the earliest cohort (68.9 vs. 20.1%, p<0.001), be older (mean age 69.7 vs 67.6, p=0.005), be White (76.1 vs. 62.6%, p<0.001), have a haemorrhagic stroke (26.6 vs. 15.8%, p<0.001), have a GCS<13 (32.9 vs. 15.6%, p<0.001), fail their swallowing test (47.6 vs. 24.0%, p<0.001), be incontinent (50.9 vs. 31.9%, p<0.001), have a post-stroke 7d BI<15 (60.5 vs. 48.2%, p=0.001), have no brain imaging done (7.9 vs. 0.5%, p<0.001), have thrombolysis (25.5 vs. 13.3%, p=0.027) and be a current smoker (46.2 vs. 29.4%, p<0.001). They are also less likely to: be admitted to a stroke unit (32.7 vs. 71.8%, p<0.001), have an MRI scan (8.1 vs. 28.1%, p<0.001), and have hypertension (54.3 vs. 66.3%, p<0.001). This suggests that those with missing outcome data have poorer functional outcomes and poorer care. Their characteristics are generally more similar to those who were discharged to a care home.

Table II. Level of missingness in each variable, stratified by outcome

Factor	Missingness (%) for discharge to:		P-value
	Care home (N=484)	Own home (N=3,441)	
Year of stroke	0 (0.0)	0 (0.0)	N/A
Age	0 (0.0)	0 (0.0)	N/A
Sex	0 (0.0)	0 (0.0)	N/A
Ethnicity	3 (0.6)	37 (1.1)	0.489
Pre-stroke living arrangement	50 (10.3)	199 (5.8)	<0.001
**Acute phase clinical characteristics**			
Stroke subtype	5 (1.0)	11 (0.3)	0.054
Glasgow coma score	22 (4.5)	81 (2.4)	0.008
Swallowing test	24 (5.0)	316 (9.2)	0.003
Incontinence	19 (3.9)	98 (2.8)	0.245
Pre-stroke Barthel Index	21 (4.3)	79 (2.3)	0.012
Post-stroke (7d) Barthel Index	51 (10.5)	344 (10.0)	0.772
NIHSS	22 (12.3)	242 (10.3)	0.484
**Acute phase process of care**			
Admission to stroke unit	3 (0.6)	3 (0.1)	0.029
Thrombolysis (if ischaemic)	1 (0.6)	14 (0.7)	0.999
Brain imaging	0 (0.0)	8 (0.2)	0.601
Length of stay	17 (3.5)	80 (2.3)	0.156
**Pre-stroke risk factors**			
Hypertension	14 (2.9)	53 (1.5)	0.050
Transient ischemic attack (TIA)	17 (3.5)	66 (1.9)	0.035
Atrial fibrillation	19 (3.9)	76 (2.2)	0.032
Myocardial infarction	18 (3.7)	88 (2.6)	0.185
Diabetes	14 (2.9)	56 (1.6)	0.074
Consumes alcohol	57 (11.8)	224 (6.5)	<0.001
Smoking status	39 (8.1)	134 (3.9)	<0.001

The other missingness of interest is that in each variable. Therefore, we looked at whether there were differences in the outcome in those with and without missing data for each variable. Those discharged to care homes had higher levels of missingness in: pre-stroke living arrangements (10.3 vs. 5.8%, p<0.001), GCS score (4.5 vs. 2.4%, p=0.008), pre-stroke BI (4.3 vs. 2.3%, p=0.012), admission to a stroke unit (0.6 vs. 0.1%, p=0.029), hypertension (2.9 vs. 1.5%, p=0.050), TIA (3.5 vs. 1.9%, p=0.035), atrial fibrillation (3.9 vs. 2.2%, p=0.032), alcohol consumption (11.8 vs 6.5%, p<0.001) and smoking status (8.1 vs. 3.9%, p<0.001) compared to those discharged to their own home. Those discharged to care homes had fewer missing data in swallowing test result results (5.0 vs. 9.2%, p=0.003) than those discharged to their own homes.

Table III. The characteristics of the whole population of stroke survivors, stratified by year of stroke

Factor	Cohort				P-value
	**1995-2000 (N=840)**	**2001-2006 (N=1215)**	**2007-2012 (N=1116)**	**2013-2018 (N=1001)**	
Age (mean (SD))	68.7 (13.9)	67.3 (15.1)	67.5 (15.8)	67.0 (15.9)	0.096
Sex: Male	434 (51.7)	655 (53.9)	606 (54.3)	560 (55.9)	0.333
Ethnicity					<0.001
White	598 (71.4)	776 (65.4)	669 (60.5)	539 (54.3)	
Black	189 (22.6)	317 (26.7)	356 (32.2)	384 (38.7)	
Other	50 (6.0)	93 (7.8)	81 (7.3)	70 (7.0)	
Living alone pre-stroke	256 (39.7)	410 (34.5)	366 (33.5)	372 (38.4)	0.017
**Acute phase clinical characteristics**					
Stroke subtype: Haemorrhagic	164 (19.9)	187 (15.4)	138 (12.4)	167 (16.7)	<0.001
Glasgow coma scale <13	121 (14.5)	162 (13.8)	194 (18.0)	155 (15.9)	0.037
Swallowing test failed	298 (36.4)	297 (27.0)	190 (19.5)	128 (14.1)	<0.001
Incontinence	324 (39.4)	416 (35.4)	295 (27.3)	256 (26.3)	<0.001
Pre-stroke Barthel Index <15	28 (3.4)	45 (3.8)	53 (5.0)	74 (7.7)	<0.001
Post-stroke (7d) Barthel Index <15	459 (64.3)	545 (48.4)	387 (40.2)	414 (43.8)	<0.001
NIHSS (median (IQR))	-	-	5.0 (3.0-10.0)	4.0 (2.0-8.2)	<0.001
**Acute phase process of care**					
Admission to a stroke unit	248 (29.6)	944 (78.0)	967 (86.6)	832 (83.1)	<0.001
Thrombolysis (if ischaemic)	-	-	164 (17.0)	110 (13.3)	0.035
Brain imaging					<0.001
No scan	14 (1.7)	3 (0.2)	1 (0.1)	1 (0.1)	
CT only	700 (84.8)	900 (74.9)	602 (62.3)	493 (63.2)	
MRI / MRI and CT	111 (13.5)	299 (24.9)	364 (37.6)	286 (36.7)	
Length of stay (days, median (IQR))	31.0 (13.0-68.0)	17.0 (7.0-50.0)	9.0 (3.0-27.0)	8.0 (3.0-25.0)	<0.001
**Pre-stroke risk factors**					
Hypertension	544 (68.3)	782 (64.7)	721 (65.4)	671 (67.6)	0.286
Transient ischaemic attack (TIA)	138 (17.3)	150 (12.4)	89 (8.2)	80 (8.1)	<0.001
Atrial fibrillation	124 (15.5)	141 (11.7)	166 (15.2)	196 (20.1)	<0.001
Myocardial infarction	91 (11.4)	108 (8.9)	98 (9.0)	119 (12.3)	0.022
Diabetes	141 (17.6)	250 (20.9)	249 (22.5)	273 (27.6)	<0.001
Consumes alcohol (>1 unit per week)	506 (66.1)	708 (61.8)	525 (50.9)	459 (49.6)	<0.001
Smoking status					<0.001
Never	285 (34.5)	430 (36.8)	418 (39.6)	410 (44.1)	
Ex-smoker	224 (27.2)	383 (32.8)	332 (31.5)	305 (32.8)	
Current	316 (38.3)	356 (30.5)	305 (28.9)	215 (23.1)	

Table IV: The associations between demographic and stroke characteristics and discharge destination post-stroke, in the 1995-2000 cohort of patients

**Factor**	**Those discharged to (%):**		**Adjusted***	
	Private home (N=548)	Care home (N=174)	OR (95% CI)	P-value
Age (mean (SD))	66.9 (14.0)	72.6 (13.4)	1.05 (1.02-1.07)	<0.001
Sex: Female	246 (44.9)	90 (51.7)	1.06 (0.64-1.74)	0.834
Ethnicity				
White	385 (70.3)	130 (74.7)	-	-
Black	128 (23.4)	36 (20.7)	0.86 (0.44-1.65)	0.647
Other	35 (6.4)	8 (4.6)	0.51 (0.15-1.52)	0.246
Pre-stroke living arrangements				
With others	251 (45.8)	88 (50.6)	-	-
Alone	170 (31.0)	63 (36.2)	0.99 (0.57-1.70)	0.965
Unknown	127 (23.2)	23 (13.2)	0.88 (0.26-2.96)	0.841
**Acute phase clinical characteristics**				
Stroke subtype: Haemorrhagic	107 (19.5)	33 (19.0)	0.82 (0.43-1.54)	0.550
Glasgow coma scale: <13	56 (10.2)	48 (27.6)	1.37 (0.73-2.57)	0.330
Swallow test: Failed	143 (26.1)	117 (67.2)	1.72 (1.01-2.94)	0.045
Incontinent	149 (27.2)	127 (73.0)	2.38 (1.37-4.14)	0.002
Pre-stroke BI: <15	13 (2.4)	9 (5.2)	1.11 (0.31-3.79)	0.872
Post-stroke (7d) BI: <15	252 (46.0)	149 (85.6)	1.19 (0.54-2.74)	0.677
Unknown	79 (14.4)	13 (7.5)	0.76 (0.19-3.05)	0.703
**Acute phase process of care**				
Admission to a stroke unit	155 (28.3)	58 (33.3)	0.90 (0.50-1.59)	0.712
Brain imaging				
CT only	453 (82.7)	157 (90.2)	-	-
MRI only/with CT	84 (15.3)	16 (9.2)	0.92 (0.38-2.11)	0.839
Unknown	11 (2.0)	1 (0.6)	0.37 (0.02-3.18)	0.434
Length of stay in hospital (days, median (IQR)	21.0 (10.0-46.0)	90.0 (61.3-117.5)	1.04 (1.03-1.05)	<0.001
**Pre-stroke risk factors**				
Hypertension	356 (65.0)	118 (67.8)	1.09 (0.64-1.87)	0.757
Unknown	23 (4.2)	5 (2.9)	1.61 (0.30-8.05)	0.569
Transient ischemic attack (TIA)	95 (17.3)	29 (16.7)	0.70 (0.36-1.35)	0.296
Unknown	19 (3.5)	6 (3.4)	1.91 (0.40-8.97)	0.411
Atrial fibrillation	77 (14.1)	24 (13.8)	0.65 (0.32-1.28)	0.218
Unknown	19 (3.5)	8 (4.6)	3.15 (0.74-13.47)	0.120
Myocardial infarction	58 (10.6)	25 (14.4)	1.21 (0.57-2.52)	0.614
Unknown	20 (3.6)	7 (4.0)	3.04 (0.68-13.02)	0.137
Diabetes	94 (17.2)	32 (18.4)	0.97 (0.49-1.87)	0.928
Unknown	18 (3.3)	5 (2.9)	4.76 (0.74-27.92)	0.090
Alcohol consumption				
<1 unit per week	165 (30.1)	62 (35.6)	-	-
>1 unit per week	340 (62.0)	102 (58.6)	0.71 (0.41-1.24)	0.234
Unknown	43 (7.8)	10 (5.7)	4.08 (0.68-35.76)	0.151
Smoking status				
Never	171 (31.2)	71 (40.8)	-	-
Ex-smoker	159 (29.0)	46 (26.4)	0.62 (0.32-1.17)	0.138
Current	218 (39.8)	57 (32.8)	0.74 (0.39-1.43)	0.370

*Adjusted for year of stroke, age, sex, ethnicity, pre-stroke living arrangement, type of stroke, stroke severity (GCS), swallow test results, incontinence, pre-stroke BI, post-stroke (7d) BI, admission to a stroke unit, brain imaging, and length of stay in hospital

Table V: The associations between demographic and stroke characteristics and discharge destination post-stroke, in the 2001-2006 cohort of patients

**Factor**	**Those discharged to (%):**		**Adjusted**	
	Private home (N=941)	Care home (N=129)	OR (95% CI)	P-value
Age (mean (SD))	66.2 (15.4)	75.3 (10.9)	1.05 (1.02-1.08)	<0.001
Sex: Female	431 (45.8)	67 (51.9)	0.62 (0.37-1.03)	0.066
Ethnicity				
White	598 (63.5)	98 (76.0)	-	-
Black	262 (27.8)	22 (17.1)	0.60 (0.30-1.15)	0.133
Other	81 (8.6)	9 (7.0)	0.80 (0.27-2.15)	0.676
Pre-stroke living arrangements				
With others	630 (67.0)	60 (46.5)	-	-
Alone	303 (32.2)	65 (50.4)	2.04 (1.22-3.46)	0.007
Unknown	8 (0.9)	4 (3.1)	6.52 (0.88-47.44)	0.069
**Acute phase clinical characteristics**				
Stroke subtype: Haemorrhagic	148 (15.7)	17 (13.2)	0.67 (0.29-1.44)	0.320
Glasgow coma scale: <13	101 (10.7)	48 (37.2)	1.89 (1.02-3.50)	0.043
Unknown	21 (2.2)	9 (7.0)	3.70 (1.15-11.35)	0.024
Swallow test: Failed	189 (20.1)	75 (58.1)	1.41 (0.79-2.50)	0.240
Not done	89 (9.5)	5 (3.9)	0.62 (0.14-2.15)	0.480
Incontinent	275 (29.2)	93 (72.1)	1.05 (0.56-1.95)	0.884
Unknown	28 (3.0)	4 (3.1)	0.58 (0.14-1.98)	0.416
Pre-stroke BI: <15	23 (2.4)	14 (10.9)	2.21 (0.91-5.35)	0.079
Unknown	9 (1.0)	3 (2.3)	1.19 (0.16-8.17)	0.861
Post-stroke (7d) BI: <15	372 (39.5)	113 (87.6)	3.60 (1.45-10.26)	0.009
Unknown	63 (6.7)	10 (7.8)	5.74 (1.71-20.14)	0.005
**Acute phase process of care**				
Admission to a stroke unit	738 (78.4)	108 (83.7)	1.15 (0.58-2.36)	0.694
Brain imaging				
CT only	684 (72.7)	118 (91.5)	-	-
MRI only/with CT	257 (27.3)	11 (8.5)	0.42 (0.17-0.97)	0.053
Length of stay in hospital (days, median (IQR))	14.0 (6.0-36.0)	82.0 (59.0-115.0)	1.03 (1.02-1.04)	<0.001
**Pre-stroke risk factors**				
Hypertension	614 (65.2)	76 (58.9)	0.63 (0.38-1.07)	0.088
Unknown	2 (0.2)	2 (1.6)	2.38 (0.14-32.15)	0.504
Transient ischemic attack (TIA)	121 (12.9)	9 (7.0)	0.30 (0.12-0.69)	0.007
Atrial fibrillation	105 (11.2)	22 (17.1)	0.99 (0.50-1.93)	0.981
Myocardial infarction	80 (8.5)	14 (10.9)	1.12 (0.47-2.52)	0.793
Diabetes	191 (20.3)	28 (21.7)	0.85 (0.46-1.53)	0.600
Unknown	11 (1.2)	2 (1.6)	0.53 (0.05-4.24)	0.561
Alcohol consumption				
<1 unit per week	337 (35.8)	52 (40.3)	-	-
>1 unit per week	557 (59.2)	67 (51.9)	0.96 (0.55-1.68)	0.880
Unknown	47 (5.0)	10 (7.8)	1.68 (0.52-5.04)	0.366
Smoking status				
Never	327 (34.8)	60 (46.5)	-	-
Ex-smoker	307 (32.6)	37 (28.7)	0.82 (0.45-1.50)	0.530
Current	278 (29.5)	26 (20.2)	0.97 (0.48-1.93)	0.941
Unknown	29 (3.1)	6 (4.7)	0.37 (0.09-1.44)	0.163

*Adjusted for year of stroke, age, sex, ethnicity, pre-stroke living arrangement, type of stroke, stroke severity (GCS), swallow test results, incontinence, pre-stroke BI, post-stroke (7d) BI, admission to a stroke unit, brain imaging, and length of stay in hospital

Table VI: The associations between demographic and stroke characteristics and discharge destination post-stroke, in the 2007-2012 cohort of patients

**Factor**	**Those discharged to (%):**		**Adjusted**	
	Private home (N=875)	Care home (N=90)	OR (95% CI)	P-value
Age (mean (SD))	65.5 (15.9)	80.3 (10.5)	1.08 (1.05-1.11)	<0.001
Sex: Female	383 (43.8)	50 (55.6)	0.67 (0.36-1.22)	0.189
Ethnicity				
White	513 (58.6)	71 (78.9)	-	-
Black	300 (34.3)	12 (13.3)	0.55 (0.25-1.18)	0.136
Other	62 (7.1)	7 (7.8)	0.58 (0.17-1.77)	0.356
Pre-stroke living arrangements				
With others	587 (67.1)	48 (53.3)	-	-
Alone	279 (31.9)	38 (42.2)	1.58 (0.86-2.89)	0.137
Unknown	9 (1.0)	4 (4.4)	2.43 (0.37-14.90)	0.340
**Acute phase clinical characteristics**				
Stroke subtype: Haemorrhagic	110 (12.6)	8 (8.9)	0.55 (0.20-1.39)	0.223
Glasgow coma scale: <13	119 (13.6)	40 (44.4)	2.07 (1.04-4.15)	0.039
Unknown	22 (2.5)	6 (6.7)	2.88 (0.64-11.97)	0.153
Swallow test: Failed	111 (12.7)	48 (53.3)	2.30 (1.16-4.57)	0.017
Not done	107 (12.2)	7 (7.8)	1.45 (0.47-4.08)	0.499
Incontinent	169 (19.3)	71 (78.9)	3.77 (1.93-7.62)	<0.001
Unknown	21 (2.4)	2 (2.2)	1.18 (0.13-6.30)	0.861
Pre-stroke BI: <15	29 (3.3)	10 (11.1)	0.54 (0.19-1.45)	0.234
Unknown	29 (3.3)	9 (10.0)	2.04 (0.64-6.16)	0.213
Post-stroke (7d) BI: <15	239 (27.3)	74 (82.2)	10.58 (3.63-40.25)	<0.001
Unknown	117 (13.4)	12 (13.3)	5.44 (1.46-23.84)	0.015
**Acute phase process of care**				
Admission to a stroke unit	772 (88.2)	80 (88.9)	0.97 (0.41-2.45)	0.949
Brain imaging				
CT only	470 (53.7)	57 (63.3)	-	-
MRI only/with CT	301 (34.4)	20 (22.2)	0.99 (0.47-2.01)	0.973
Unknown	104 (11.9)	13 (14.4)	3.43 (1.36-8.53)	0.008
Length of stay in hospital (days, median (IQR))	7.0 (2.0-20.0)	47.0 (32.5-71.75)	1.01 (1.01-1.02)	<0.001
**Pre-stroke risk factors**				
Hypertension	556 (63.5)	66 (73.3)	1.09 (0.57-2.14)	0.799
Transient ischemic attack (TIA)	69 (7.9)	6 (6.7)	1.05 (0.33-2.94)	0.935
Unknown	6 (0.7)	1 (1.1)	0.53 (0.01-10.95)	0.699
Atrial fibrillation	106 (12.1)	30 (33.3)	2.20 (1.10-4.42)	0.026
Myocardial infarction	77 (8.8)	9 (10.0)	0.90 (0.34-2.20)	0.822
Unknown	19 (2.2)	1 (1.1)	0.60 (0.02-5.47)	0.691
Diabetes	197 (22.5)	12 (13.3)	0.58 (0.24-1.29)	0.196
Alcohol consumption				
<1 unit per week	392 (44.8)	36 (40.0)	-	-
>1 unit per week	430 (49.1)	36 (40.0)	1.19 (0.63-2.26)	0.601
Unknown	53 (6.1)	18 (20.0)	2.93 (1.11-7.81)	0.030
Smoking status				
Never	324 (37.0)	38 (42.2)	-	-
Ex-smoker	263 (30.1)	25 (27.8)	1.06 (0.52-2.14)	0.869
Current	252 (28.8)	13 (14.4)	0.63 (0.25-1.47)	0.292
Unknown	36 (4.1)	14 (15.6)	3.48 (1.17-10.35)	0.024

*Adjusted for year of stroke, age, sex, ethnicity, pre-stroke living arrangement, type of stroke, stroke severity (GCS), swallow test results, incontinence, pre-stroke BI, post-stroke (7d) BI, admission to a stroke unit, brain imaging, and length of stay in hospital

Table VII: The associations between demographic and stroke characteristics and discharge destination post-stroke, in the 2013-2018 cohort of patients

**Factor**	**Those discharged to (%):**		**Adjusted**	
	Private home (N=806)	Care home (N=39)	OR (95% CI)	P-value
Age (mean (SD))	66.3 (15.9)	75.4 (13.7)	1.04 (1.01-1.07)	0.022
Sex: Female	355 (44.0)	15 (38.5)	0.30 (0.12-0.69)	0.007
Ethnicity				
White	442 (54.8)	23 (59.0)	-	-
Black	304 (37.7)	13 (33.3)	0.92 (0.38-2.15)	0.854
Other	60 (7.4)	3 (7.7)	0.97 (0.14-4.53)	0.975
Pre-stroke living arrangements				
With others	500 (62.0)	17 (43.6)	-	-
Alone	289 (35.9)	19 (48.7)	1.19 (0.52-2.73)	0.679
Unknown	17 (2.1)	3 (7.7)	7.61 (1.23-38.14)	0.018
**Acute phase clinical characteristics**				
Stroke subtype: Haemorrhagic	141 (17.5)	4 (10.3)	0.12 (0.02-0.54)	0.014
Glasgow coma scale: <13	120 (14.9)	14 (35.9)	0.83 (0.30-2.21)	0.718
Swallow test: Failed	85 (10.5)	18 (46.2)	2.43 (0.90-6.57)	0.078
Not done	65 (8.1)	3 (7.7)	0.94 (0.11-5.57)	0.953
Incontinent	185 (23.0)	27 (69.2)	2.24 (0.79-6.61)	0.132
Unknown	21 (2.6)	2 (5.1)	5.86 (0.60-37.34)	0.084
Pre-stroke BI: <15	56 (6.9)	9 (23.1)	2.08 (0.66-6.37)	0.202
Unknown	18 (2.2)	2 (5.1)	1.49 (0.13-11.45)	0.720
Post-stroke (7d) BI: <15	307 (38.1)	32 (82.1)	1.65 (0.44-6.99)	0.469
Unknown	34 (4.2)	3 (7.7)	3.70 (0.41-26.48)	0.211
**Acute phase process of care**				
Admission to a stroke unit	686 (85.1)	36 (92.3)	1.40 (0.30-9.49)	0.697
Brain imaging				
CT only	390 (48.4)	24 (61.5)		
MRI only/with CT	238 (29.5)	8 (20.5)	0.99 (0.33-2.74)	0.992
Unknown	178 (22.1)	7 (17.9)	0.61 (0.18-1.78)	0.387
Length of stay in hospital (days, median (IQR))	7.0 (2.0-21.0)	64.0 (47.5-90.0)	1.03 (1.02-1.04)	<0.001
**Pre-stroke risk factors**				
Hypertension	548 (68.0)	23 (59.0)	0.42 (0.18-0.99)	0.046
Transient ischemic attack (TIA)	65 (8.1)	4 (10.3)	1.08 (0.21-3.99)	0.917
Atrial fibrillation	154 (19.1)	12 (30.8)	0.85 (0.33-2.05)	0.718
Unknown	21 (2.6)	2 (5.1)	0.48 (0.05-3.17)	0.487
Myocardial infarction	96 (11.9)	5 (12.8)	0.59 (0.17-1.72)	0.364
Diabetes	226 (28.0)	12 (30.8)	0.83 (0.34-1.94)	0.674
Alcohol consumption				
<1 unit per week	390 (48.4)	17 (43.6)	-	-
>1 unit per week	366 (45.4)	12 (30.8)	1.07 (0.41-2.78)	0.896
Unknown	50 (6.2)	10 (25.6)	2.20 (0.71-6.56)	0.162
Smoking status				
Never	341 (42.3)	14 (35.9)	-	-
Ex-smoker	253 (31.4)	7 (17.9)	0.68 (0.21-2.03)	0.493
Current	166 (20.6)	9 (23.1)	2.41 (0.75-7.78)	0.135
Unknown	46 (5.7)	9 (23.1)	1.62 (0.51-4.94)	0.401

*Adjusted for year of stroke, age, sex, ethnicity, pre-stroke living arrangement, type of stroke, stroke severity (GCS), swallow test results, incontinence, pre-stroke BI, post-stroke (7d) BI, admission to a stroke unit, brain imaging, and length of stay in hospital
