# Supplementary material for: Long-Term Trends in Stroke Survivors Discharged to Care Homes: The South London Stroke Register
Source: Stroke. 2019 Nov 6;51(1):179–85. doi: 10.1161/STROKEAHA.119.026618 (PMC6924949; doi:10.1161/STROKEAHA.119.026618)
Supplement: Supplementary file 2 [file str-51-179-s002.pdf]

Stroke Journal <stroke@strokeahajournal.org>

RE: STROKE/2019/026618R1 Decision Letter

Clery, Amanda <amanda.clery@kcl.ac.uk> Fri, Sep 13, 2019 at 9:24 AM
To: Stroke Editorial Office <stroke@strokeahajournal.org>

Dear Erinn,

We have been able to contact the family. I have an email address for Chris' husband, his legal designee: terrydesign@hotmail.com

Please send Chris' form to him, and please could you also confirm that his login details will not be required?

Let me know if you need further information from me.

Thanks,
Amanda

From: Stroke Editorial Office <stroke@strokeahajournal.org>
Sent: 04 September 2019 13:43:55
[Quoted text hidden]

[Quoted text hidden]
