# Supplementary material for: Long-Term Trends in Stroke Survivors Discharged to Care Homes: The South London Stroke Register
Source: Stroke. 2019 Nov 6;51(1):179–85. doi: 10.1161/STROKEAHA.119.026618 (PMC6924949; doi:10.1161/STROKEAHA.119.026618)
Supplement: Supplementary file 3 [file str-51-179-s003.pdf]

**From:**	Clery, Amanda
**To:**	Erinn Cain
**Subject:**	Re: Manuscript Correspondence STROKE/2019/026618R2
**Date:**	Monday, October 7, 2019 11:38:12 AM

*** **CAUTION:** This email originated from outside of the **American Heart Association**. Do **not** click links or open attachments unless you recognize the sender and know the content is safe. ***

Dear Erinn,

Yes I can confirm that Prof. McKevitt conceived and designed the research, handled funding for the research and made critical revisions to the manuscript.

Thanks,
Amanda

From: Erinn.Cain@heart.org <Erinn.Cain@heart.org>
Sent: 07 October 2019 16:33
To: Clery, Amanda <amanda.clery@kcl.ac.uk>
Subject: Manuscript Correspondence STROKE/2019/026618R2

MS No.: STROKE/2019/026618R2
MS Title: Long-term trends in stroke survivors discharged to care homes: The South London Stroke Register

Dear Dr. Clery,

Before I send your manuscript to production, there is one item that needs to be addressed: As Prof. McKevitt's license agreement form was completed by his designee, could you please provide confirmation that Prof. McKevitt fulfilled authorship criteria? You may do so by replying to this email.

The criteria are outlined on the license agreement form.

Thank you,

Erinn Cain
Stroke

Stroke
Editorial Office

Please click on the link below to log in to the system.

https://stroke-submit.aha-journals.org/cgi-bin/main.plex?

el=A5Hn6CFj7A7Dtpp1J3A9ftdtg1pSELJ30A9Xn7e9ATZnAZ
